# On Whole Setts of Teeth

**Published:** 1848-01

**Authors:** W. E. Ide


For the Dental Register.

### ON WHOLE SETTS OF TEETH.

BY W. E. IDE, M. D.

*No.* 1.

Though for a long time rude attempts have been made, a few years only have elapsed since the discovery of any method for constructing an entire artificial denture of such perfection as to be to any considerable extent a substitute for the natural organs. As it is my design to make the following remarks entirely practical, I shall not delay to notice the various dental appliances that have become obsolete, or to mention *all* the names of those to whom the profession is indebted for the various improvements which now enable the skillful and well-informed practitioner to approach so near the long desired goal—the *Ultima Thule* of our art. It is no part of my present design to show the *failures* from which have resulted our present *successes*, or to eulogise those who have been the *avant coureurs* in the path of discovery. My only aim will be to explain briefly the method which I approve and practice, in constructing entire setts of artificial teeth.

Many of the items in my method will be found identical with those common to the majority of dental practitioners; some, perhaps, may have been practiced only by a few, and in one or two particulars it will be seen that I differ from all.

As our science is comparatively a new one, and as consequently, we have been so much indebted to oral teachings—the practice

* If the foil be adhesive the cutting of it will unite the edges of the strips, so as to appear as but one thickness. If it be wanting in that quality it will shake apart like so many folds of paper, and cannot be used in this manner, and is hardly fit for use at all.

of no two individuals in the profession is precisely the same in detail, though all aim at and may obtain, the same results.

We will first make a few suggestions in reference to a preparation of the mouth, for an artificial denture. A difference of opinion, or at least of practice, seems to exist in reference to the *time* that should intervene between the extraction and dental replacement. In regard to this, it is impossible to be governed by any fixed rules, as no two cases will be found exactly alike. Osseous absorption is well known to be more rapid in a young, than in an old patient, and in those of one temperament, than in those of another. Again—we find in nearly all old patients the alveolar ridge has been partially absorbed before the teeth were extracted. Indeed, in many instances this has been the entire cause of the loss, or removal of the teeth.

In cases of this kind, of course less time is required in preparing the mouth than when the entire alvioli have to be removed after extraction. In many instances much observation and experience is required to enable us to judge with accuracy, in regard to the time the artificial denture may with safety be applied. In no case should less than two months elapse after the extraction of the last teeth, and in many instances a whole year is required.

Many persons are too impatient, to wait a sufficient length of time, and in these cases a temporary sett may be applied in one or two weeks after extraction, and worn till the jaws have assumed a permanent shape. No consideration should induce the dentist to risk his reputation by inserting a plate designed to be permanent, before there is a perfect certainty that all change has ceased in the alveolar ridge—for the least failure in adaptation must mar, to a greater or less degree, the success of the operation.

To hasten the process of absorption, the patient should be directed to use friction upon the gums by the fingers several times in a day, and an astringent wash to remove the irritability of the mucous membranes.

After the mouth is thoroughly prepared, the next process is to take an impression in wax of the jaws. This operation requires a tact and dexterity that are only acquired to perfection, by much practice, a natural delicacy of touch, and a mechanical eye.

No skill in the operator can ensure success when this part of the operation is bunglingly, or imperfectly performed—and from no other source comes so many failures in the final result.

For this purpose the best of yellow wax should be procured. A great proportion of that found in commerce is adulterated with tallow, which destroys its tenacity and renders it entirely unfit for our purpose. The bad article may be readily detected by its being of a *pale* yellow, instead of a bright straw color. For convenience, it should be prepared and laid aside in plates about the thickness of stout leather, ready for use. It has then only to be warmed over a fire or spirit lamp and worked in the fingers till every part is equally soft—taking care that it be neither so stiff as to require too much pressure to produce an impression, or so soft as to bend too readily in removing from the mouth.

Various patterns of wax-holders are used, but the one that I prefer is made of thick plate tin. They should be of various sizes and are made by stamping the tin between the dies that have been used for previous setts of teeth. By a little trouble at the time of making each new job, a piece of tin shaped like the gold plate can be stamped and laid aside, till a sufficient variety is obtained to suit almost any case. For convenience in handling, a bit of tin tube about a half inch in diameter and two inches long can be soldered to the front part, and the instrument is complete. In cases where there is difficulty in getting a perfect impression, and there has been a failure in the fit, I often use the gold plate that has been stamped from the imperfect dies; or, if a handle is desired a tin plate is prepared instead of the gold, and the handle is soldered on as in other cases. This, of course, approximates so near a fit, that but a small quantity of wax is necessary for a second impression, and the plate being pressed down firmly upon the gums, the slightest care will secure a perfect impression.

The wax and the holder being prepared, are introduced into the mouth, taking care to bring the lips forward out of the way.

In pressing it upon the gums, a finger of each hand should be placed on each side of the wax-holder, and the force applied as directly and equally as possible. It is then to be held firmly in its position by one hand, while the fingers of the other are used in pressing the wax to the gums in front of the arch, and upon the hard palate.

When the arch is very prominent, the suction is often so great as to render it very difficult to remove the wax without spoiling the impression. The lips also are often so contracted as to increase this difficulty, and great care and tact are necessary to ensure success.

Another method was communicated to the American Society of Dental Surgeons at their sixth annual meeting by Dr. Bridges. He uses plaster of paris instead of wax, in the following manner:—

A rim of softened wax is placed on every side of the common wax holder, and plaster of paris, mixed to the consistence of batter, is poured into it, and this is pressed upon the gums and held there from three to five minutes, or until it becomes so solid as not to break in the removal. This is then oiled or varnished, and used precisely as the wax impression described above. A perfect impression may undoubtedly be procured in this way, but as it must evidently be objectionable to the patient, we cannot recommend it, except in cases where other methods have failed.

Perhaps the method most certain of all, and in many cases the least troublesome, is that practiced by M. Desirabode.*

He takes an impression in wax as perfect as possible, and then, after getting up the metallic dies, as will be hereafter described, he stamps with them a plate of lead of the shape desired for the plate of gold. If, on applying this to the mouth, it is found to be a perfect fit, it is thrown aside—if not, it is adapted to the alveolar arch by the fingers, and being carefully removed, is used for getting up a new plaster cast, precisely as was the wax impression. I have been thus minute in describing this part of the operation for the reason that any imperfection here cannot fail to spoil all that may be done subsequently, no matter how great may be the skill of the Dentist. In the next number we will describe the method of getting up the various dies, articulating models, the stamping of the plate, the arranging of the teeth, &c.

* Vide Nauveaux Elements Complets de la Science et de l'art du Dentiste, t. 2, p. 636.

